# Delayed breast cancer diagnosis after repeated recall at biennial screening mammography: an observational follow-up study from the Netherlands

**DOI:** 10.1038/s41416-020-0870-2

**Published:** 2020-05-11

**Authors:** Joost R. C. Lameijer, Adri C. Voogd, Ruud M. Pijnappel, Wikke Setz-Pels, Mireille J. Broeders, Vivianne C. G. Tjan-Heijnen, Lucien E. M. Duijm

**Affiliations:** 1 Department of Radiology, Catharina Hospital Eindhoven, Michelangelolaan 2, 5623 EJ Eindhoven, The Netherlands; 2 Department of Internal Medicine, Division of Medical Oncology, GROW, Maastricht University Medical Centre, P Debyelaan 1, 6229 HA Maastricht, The Netherlands; 3 Department of Epidemiology, GROW, Maastricht University, P Debyelaan 1, 6229 HA Maastricht, The Netherlands; 4 Department of Research, Netherlands Comprehensive Cancer Organization (IKNL), Godebaldkwartier 419, 3511 DT Utrecht, The Netherlands; 5 Department of Radiology and Nuclear Medicine, University Medical Center Utrecht, Utrecht University, Heidelberglaan 100, 3584 CX Utrecht, The Netherlands; 6 Dutch Expert Centre for Screening, Wijchenseweg 101, 6538 SW Nijmegen, The Netherlands; 7 Department for Health Evidence, Radboud University Medical Center, P.O. Box 9101, 6500 HB Nijmegen, The Netherlands; 8 Department of Radiology, Canisius Wilhelmina Hospital, Weg door Jonkerbos 100, 6532 SZ Nijmegen, The Netherlands

**Keywords:** Breast cancer, Population screening, Epidemiology

## Abstract

**Background:**

Delay in detection of breast cancer may worsen tumour characteristics, with progression of tumour size and a higher risk of metastatic lymph nodes. The purpose of this study was to investigate delayed breast cancer diagnosis after repeated recall for the same mammographic abnormality at screening.

**Methods:**

This was a retrospective study performed in two cohorts of women enrolled in a mammography screening programme in the Netherlands. All women aged 50−75 who underwent biennial screening mammography either between January 1, 1997 and December 31, 2006 (cohort 1) or between January 1, 2007 and December 31, 2016 (cohort 2) were included.

**Results:**

The cohorts showed no difference in proportions of women with delayed breast cancer diagnosis of at least 2 years (2.2% versus 2.8%, *P* = 0.29). Most delays were caused by incorrect BI-RADS classifications after recall (74.2%). An increase in mean tumour size was seen when comparing sizes at initial false-negative recall and at diagnosis of breast cancer (*P* < 0.001).

**Conclusions:**

The proportion of women with a long delay in breast cancer confirmation following repeated recall at screening mammography has not decreased during 20 years of screening. These delays lead to larger tumour size at detection and may negatively influence prognosis.

## Background

The Dutch nationwide biennial screening mammography programme has been shown to be effective in the early detection of breast cancer and reduction in breast cancer mortality.^1,2^ Early detection of breast cancer does not solely involve detection of suspicious mammographic abnormalities at screening, as a swift confirmation of malignant lesions after recall is mandatory as well for the success of the screening programme.

Delay in the detection of breast cancer remains an important topic in health care, both for its frequency of occurrence and its possible negative effects on survival.^3–8^ A delay in breast cancer diagnosis may worsen tumour characteristics, with a progression of tumour size and a higher risk of metastatic lymph nodes. Previous studies have mostly focused on shorter diagnostic delays in symptomatic patients and very limited data are available on this subject in asymptomatic, screen-detected breast cancers.^3,5,6,8^ Ciatto et al.^9^ found that 1.4% of women experienced a delay of at least 2 years in their breast cancer confirmation after repeated recall for a screen-detected abnormality. Several causes for a delayed breast cancer diagnosis after recall at screening have been reported, including an improper classification of mammographic abnormalities at subsequent clinical breast imaging, communication errors between physicians, and sample errors at biopsy.^3,6,9^

The last decade is characterised by substantial changes in the diagnostic management of (a)symptomatic breast disease, including the introduction of multidisciplinary meetings and new imaging modalities (e.g., digital breast tomosynthesis), an intensified use of advanced imaging modalities (breast MRI), a further replacement of fine-needle biopsy by (vacuum-assisted) large core biopsy, and an increased sub-specialisation of physicians in breast care.^6^ However, the impact of these improvements on the proportion of women who experience a long delay in breast cancer diagnosis after recall at screening mammography is unknown. In the current study, we focused on women who experienced a delay of at least 2 years in their confirmation of breast cancer following a repeated recall for the same mammographic abnormality at biennial screening mammography in the period from 1997 to 2016. We investigated this delay over time, comparing two screening cohorts of 10 years each, and specified the radiological and tumour characteristics of the cancers with a diagnostic delay. Finally, we determined the causes of diagnostic delay and assessed whether the hospitals that handled recall varied in the number of women who experienced a delay in their breast cancer confirmation.

## Methods

### Study population and screening procedure

This is a retrospective observational study, based on a prospective database of women aged 50−75 years attending the biennial breast cancer screening programme conducted in the south of the Netherlands. Characteristics of this programme have been reported previously.^10–12^ In brief, women are personally invited by letter to attend the programme, with about 80% of the women accepting the invitation. Women who are being treated for breast cancer and those who still receive clinical oncologic follow-up after breast cancer treatment usually do not attend the screening programme.

A consecutive series of 283,479 screen-film mammography screens (39,098 initial screens and 244,381 subsequent screens) were included between January 1, 1997 and December 31, 2006 and this series is labelled cohort 1. A second consecutive series of 534,177 screen-film and full-field digital mammography (FFDM) screens (58,443 initial screens and 475,734 subsequent screens) were included between January 1, 2007 and December 31, 2016 and this series is labelled cohort 2.

Screen-film mammograms were obtained with commercially available units (Performa, Oldelft, Tuusula, Finland) and dedicated mammography screens were utilised (Mamoray MR-R; Agfa, Mortsel, Belgium). Dedicated film was used (Mamoray HDR; Agfa, Mortsel, Belgium) to process mammography screens and processing was done by extended-cycle dedicated processing. During 2009/2010, screen-film mammography in the southern screening region of the Netherlands was gradually replaced by FFDM. All digital mammograms were obtained with a Lord Selenia FFDM system (Hologic Inc, Danbury, CT), with a 70 µm pixel size and a 232 × 286 mm field of view.

The examinations were obtained by specialised screening mammography radiographers. Since the start of FFDM screening in 2009/2010, two-view mammography (medio-lateral-oblique (MLO) and cranio-caudal (CC) view) of each breast was performed in first as well as subsequent screens. In the era of screen-film screening, all women who attended the programme for the first time received two-view mammography, whereas subsequent screens comprised a routine MLO view of each breast and additional CC views if indicated. All screening mammograms were then independently double read by certified breast cancer screening radiologists. Since the start of our screening programme in 1995, all previous screens were always available for comparison at the time of assessment of a new screening examination. Screening radiologists categorised abnormal mammographic findings into one of the following categories: suspicious mass, suspicious calcifications, suspicious mass combined with calcifications, asymmetry, architectural distortion, or other abnormalities not otherwise categorised. In the FFDM screening period only, each recall was also classified according to the Breast Imaging Reporting and Data System (BI-RADS) lexicon and the radiologists annotated each recalled mammographic abnormality on a drawing which was part of the recall report.^13,14^ Women with a BI-RADS 1 (normal findings) or 2 (benign findings) were not recalled. Women with a BI-RADS 0, 4 or 5 finding were referred to a dedicated breast unit of a hospital for further analysis. BI-RADS category 3 (probably benign findings) is not used in the Dutch screening programme, as short-term follow-up is not available in the screening setting.

### Diagnostic workup after recall

The diagnostic workup in the vast majority of recalled women (98%) was performed in six regional hospitals in the south of the Netherlands, whereas the remainder underwent additional examinations at various other hospitals. After physical examination by a surgical oncologist or dedicated breast nurse, additional breast imaging (when needed) was performed at the radiology department. The screening mammogram was first reassessed by a radiologist and the screening mammograms were routinely available. Additional mammographic projections were obtained at the discretion of the radiologist. Breast ultrasonography was used for the additional characterisation of mammographic abnormalities and palpable breast lesions, as guidance for biopsy and for target or second look purposes following breast MRI. Breast MRI gradually became available in the hospitals from 1998 while whole breast ultrasound was performed in only one of the six hospitals. Digital breast tomosynthesis was introduced in 2011 and available in all but one of the hospitals in the later years of inclusion. Fine-needle aspiration biopsy (FNAB) and percutaneous core needle biopsy (CNB) were available in each hospital from the beginning of our study and FNAB was gradually replaced by CNB in the 1990s. Vacuum-assisted stereotactic core needle biopsy (SCNB) was introduced in 2000 and was available in each hospital from 2004. Multidisciplinary meetings for the discussion of recalled women were gradually implemented in the hospitals.

During a minimum of 2-year follow-up, clinical data and data from diagnostic breast imaging, biopsy specimen and surgical procedures were collected of all recalled women by one of the screening radiologists (L.E.M.D.) and several radiology residents. The radiologist then entered these data in a database that had been constructed for quality assurance of the screening programme in the south of the Netherlands. Breast cancers were categorised into ductal carcinoma in situ (DCIS) and invasive cancers. Lobular carcinoma in situ was considered a non-malignant lesion. The TNM classification (6th and 7th edition) was used for malignant lesions.^15,16^ For all cancers treated by neo-adjuvant therapy (either chemotherapy or endocrine therapy), the initial tumour size was derived from breast imaging (usually MRI) prior to the start of this therapy.

### Delay

In this study, we focused on women diagnosed with breast cancer after a second recall for a mammographic abnormality, which had been considered benign at workup after the first (initial) recall. Therefore, the delay in breast cancer diagnosis in these women was at least 2 years. We limited our study to these delays because this allowed us to review the initial and the subsequent screening mammograms to measure tumour size and calculate progression. As we had only access to the radiology reports and the screening mammograms and not to the mammographic images obtained after recall, we could not determine tumour sizes at the clinical mammograms of women with a diagnostic delay of less than 2 years following recall.

The database for quality assurance was used to identify women who had been recalled twice between January 1997 and January 2017 and two radiologists (L.E.M.D., W.S.-P.) then determined independently whether or not the second recall concerned the same lesion for which a woman had been recalled previously. Discrepant observations between the two radiologists were solved by consensus. For all cases in which both recalls concerned the same mammographic abnormality and the second recall resulted in confirmation of invasive breast cancer, the two radiologists measured the diameter of the suspicious abnormality both at the initial, false-negative recall, and at the second, true-positive recall. These measurements were also done in an independent fashion, followed by consensus reading. Both reviewers knew they reassessed cases characterised by a delay in breast cancer diagnosis. We restricted the reassessment of tumour sizes on the screening mammograms to cohort 2 as, contrary to cohort 1, almost all screening examinations of cohort 2 consisted of two-view mammography which better enabled the radiologists to determine lesion size. Like the screening radiologists, the two radiologists had the availability of all previous screening rounds at the time they reviewed women with a possible delay in cancer diagnosis. To explore the causes of the diagnostic delays, the radiologists reviewed the clinical data (including radiological findings and biopsy reports, discharge records, outcome of multidisciplinary team meetings) and assessed whether a delay was due to an erroneous BI-RADS classification assignment at workup, a false-negative biopsy result, a patient related delay, or other causes.

### Ethical approval and informed consent

Ethical approval by our local Institutional Review Board was not required for this observational follow-up study, according to the Dutch Central Committee on Research involving Human Subjects (CCMO). Prior to their participation in the programme, women were asked for permission to use their data for the evaluation of the screening programme and scientific purposes.

### Statistical analysis

Descriptive statistics were performed using Statistical Package for Social Science 23.0 (SPSS Inc., IBM, Chicago, IL). The chi-square test was used to test for differences between cohorts with respect to categorical data and for differences between hospitals with respect to delayed breast cancer diagnoses in cohort 2. A *P* value < 0.05 was considered to indicate a statistically significant difference. *P* values were two-sided. Whenever applicable (due to small sample sizes), the Fisher’s exact test was used.

The dependent samples *T* test was used to compare the mean tumour size of invasive cancers in cohort 2 at the false-negative assessment after initial recall with the mean tumour size at subsequent (second) recall when breast cancer was finally confirmed. To test for differences in median delay between the two cohorts, the Mann−Whitney *U* test was used. Similarity in distributions of both cohorts with respect to delays was tested and comparable distributions were found. Data in cohort 2 were missing in <0.5% of the patients, which in most cases involved the lack of oestrogen or progesterone receptor status due to an insufficient tissue sample. In the analysis of the categorical data, we treated the missing numbers as a separate category to allow a complete case analysis.

## Results

### Cohort characteristics

The recall rate was significantly higher in cohort 2 (2.8% (15,145/534,177) versus 1.2% (3447/283,479), *P* < 0.001, Table 1). Compared to the first cohort, this higher recall rate in cohort 2 came along with a higher cancer detection rate (6.6 versus 5.0 per 1000 screens, *P* < 0.001) and a lower positive predictive value (PPV) of recall (23.2% versus 41.2%).

**Table 1:** Overall screening outcome of women screened between 1997−2006 and 2007−2016.

Screening years	1997−2006	2007−2016	*P* value
Screens, *N*	283,479	534,177	
First screens, *N* (%)	39,098 (13.8)	58,443 (10.9)	
Subsequent screens, *N* (%)	244,381 (86.2)	475,734 (89.1)	
Recall, *N* (%)	3447 (1.2)	15,145 (2.8)	<0.001
Screen-detected cancers, *N*	1421	3511	<0.001
Cancer detection rate^a^	5.0	6.6	<0.001
PPV of recall, %	41.2	23.2	<0.001
Delayed breast cancer diagnosis, *N* (%)^b^	32 (2.2)	98 (2.8)	0.29
Delay in months (range)	45.0 (24 – 97)	33.8 (21 – 97)	0.001

*PPV* positive predictive value.

^a^Per 1000 screens.

^b^Proportion as the number of diagnostic delays among all screen-detected breast cancers.

### Frequency of delayed breast cancer confirmation and tumour characteristics of invasive cancers with a diagnostic delay

Focusing on a delay in breast cancer diagnosis of at least 2 years after the first recall, 2.2% (32/1421 women) who were diagnosed with breast cancer in cohort 1 experienced this delay, compared to 2.8% (98/3,511) in cohort 2 (*P* = 0.29). The median delay in cohort 1 was 26.0 months (range 24−97 months), whereas the median delay in cohort 2 was 25.0 months (range 24−97 months). The difference in median delay of the two cohorts was not statistically significant (*P* = 0.12). In cohort 1, 12 out of the 32 women (37.5%) with a diagnostic delay had their breast cancer confirmed at the next biennial screen, 2 years after their first, false-negative recall. In the remaining 20 women, breast cancer was finally confirmed after a second recall that took place at least 4 years after the first recall. In cohort 2, 66.3% of women (65/98) with a diagnostic delay had their breast cancer confirmed at the next biennial screen (37.5% versus 66.3%, *P* = 0.003).

Sufficient data in cohort 2 were available to compare the tumour characteristics of breast cancers with or without a diagnostic delay. A smaller proportion of DCIS was found among breast cancers with a delayed diagnosis (9.2% (9/98) versus 20.8% (710/3414), *P* = 0.005, Table 2). Tumour histology of invasive cancers also differed between the two groups, with a lower proportion for invasive ductal cancers and more invasive cancers of “other” type (e.g., tubular cancers, mucinous cancers) among the cases with a diagnostic delay (*P* = 0.006).

**Table 2:** Tumour characteristics of breast cancers with or without a diagnostic delay following recall in cohort 2.

	Delay in breast cancer diagnosis (*N* = 98)^a^	No delay in breast cancer diagnosis (*N* = 3413)	*P* value
Type of cancer, *N* (%)			0.005
DCIS	9 (9.2)	710 (20.8)	
Invasive	89 (90.8)	2703 (79.2)	
Histology of invasive cancers, *N* (%)			
Ductal	64 (71.9)	2137 (79.0)	0.006
Lobular	11 (12.4)	322 (11.9)	
Ductolobular	1 (1.1)	101 (3.7)	
Other	13 (14.6)	143 (5.3)	
Tumour stage of invasive cancers, *N* (%)			
T1a + b	32 (35.9)	945 (35.0)	0.32
T1c	40 (44.9)	1196 (44.2)	
T2+	17 (19.2)	558 (20.6)	
Unknown	0	4 (0.1)	
Lymph node status of invasive cancers, *N* (%)			
N+	13 (14.6)	614 (22.7)	0.14
N−	75 (84.3)	2023 (74.9)	
Unknown	1 (1.1)	66 (2.4)	
Bloom & Richardson grade, *N* (%)			
I	38 (42.7)	1213 (44.9)	0.19
II	39 (43.8)	1145 (42.4)	
III	11 (12.4)	308 (11.4)	
Unknown	1 (1.1)	37 (1.4)	
Oestrogen receptor status, *N* (%)			
Positive	82 (92.1)	2429 (89.9)	0.80
Negative	7 (7.9)	259 (9.6)	
Unknown	0	15 (0.6)	
Progesterone receptor status, *N* (%)			
Positive	57 (64.0)	1 943 (71.9)	0.16
Negative	32 (36.0)	737 (27.3)	
Unknown	0	23 (0.9)	
Her2/Neu receptor status, *N* (%)			
Positive	6 (6.7)	262 (9.7)	0.81
Negative	83 (93.3)	2415 (89.3)	
Unknown	0	26 (1.0)	
Triple-negative receptor status, *N* (%)			
Yes	5 (5.6)	173 (6.4)	0.99
No	84 (94.4)	2515 (93.0)	
Unknown	0	15 (0.6)	
Final surgical treatment, *N* (%)			
Breast conserving surgery	78 (79.5)	2756 (80.8)	0.74
Mastectomy	18 (18.4)	616 (18.0)	
No surgery	2 (2.1)	41 (1.2)	

*DCIS* ductal carcinoma in situ.

^a^Diagnostic delay was defined as confirmation of breast cancer ≥ 24 months following initial recall.

Tumour size of invasive cancers was comparable for both groups, with a majority of cancers sized between 10 and 20 mm (45.5% of invasive cancers with a delayed diagnosis versus 44.2% of properly diagnosed invasive cancers, *P* = 0.33). Invasive cancers also showed a similar histological grade distribution in both groups, with a majority of them classified Bloom & Richardson grade I or II (87.6% (78/89) versus 87.2% (2358/2703), *P* = 0.19). The majority of invasive breast cancers were lymph node negative in both groups, although there was a slightly larger number of lymph-node-negative cancers in case of a delayed diagnosis (84.3% (75/89) versus 74.9% (2023/2703), *P* = 0.14). We observed no significant differences in oestrogen-receptor, progesterone-receptor and Her2/Neu receptor status, as well as triple-negative receptor status, between the two groups. Finally, the type of surgical therapy (breast conserving surgery or mastectomy) was also similar for both groups (*P* = 0.74).

### Causes of delay in breast cancer confirmation

**Fig. 1:**
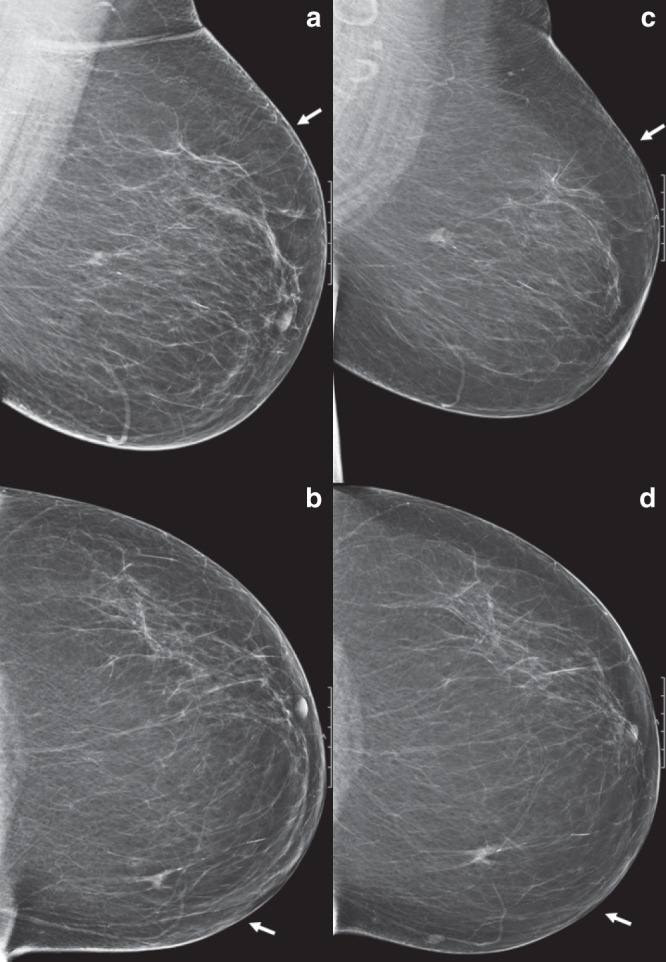
Patient example, repeated recall after previous false-negative recall. Two-view screening mammograms (**a** and **c**, medio-lateral oblique (MLO) view, and **b** and **d**, cranio-caudal (CC) view) of the left breast in 2014 (**a** and **b**) and in 2016 (**c** and **d**). In 2014, it shows a lesion in the medial upper quadrant of the breast (arrows), initially classified as BI-RADS 0 (additional analysis needed) by the screening radiologist. At recall (2014), additional digital breast tomosynthesis (DBT) and ultrasound were performed, and the lesion was classified as BI-RADS 2 (benign lesion). No biopsy was performed. Two years later (2016), the patient was recalled for the same lesion now classified as BI-RADS 5 (due to spiculae). Ultrasound-guided true-cut biopsy was performed, which revealed an invasive lobular carcinoma without axillary metastases. The patient was treated with breast conserving surgery and adjuvant radiotherapy.

In cohort 1, a false-negative biopsy (13 cases, 40.6%) was the most frequent cause of a diagnostic delay after the first recall, followed by an erroneous BI-RADS classification given by the clinical radiologists after the first recall (10 cases, 31.3%). The latter women received a BI-RADS 1 (no abnormalities) or BI-RADS 2 (benign) classification without additional biopsy. Another seven lesions (21.9%) were classified as BI-RADS 3 at initial assessment and follow-up consisted of imaging only. These seven lesions proved to be malignant after the second recall of these lesions at a subsequent screening round. In one case the surgeon did not follow the radiologists’ advice for additional biopsy and one woman refused biopsy of a suspicious mass. In cohort 2, a majority of the diagnostic delays (73 cases, 74.5%) was attributed to an erroneous BI-RADS classification after the first recall. A false-negative biopsy after the first recall was observed in 18 cancers (18.4%) with a delayed diagnosis. In 17 of these women (17/18, 94.4%), the false-negative biopsy was due to a sample error as repeated biopsy at subsequent recall 2 years later yielded proof of malignancy. In one case, the patient refused a repeated biopsy of a lesion that had grown at follow-up. Another six lesions (6.1%) were classified as BI-RADS 3 at initial assessment and one patient (1.0%) underwent excision of a lesion other than the recalled one and the latter lesion proved malignant after the second recall. An example of delayed breast cancer diagnosis due to previous false negative recall is shown in Fig. 1.

### Tumour size at screening mammography of cancers with a diagnostic delay

The tumour size in patients with a delayed diagnosis of invasive breast cancer was measured, both at the screening mammogram that initiated the first, false-negative recall and at the screening mammogram that resulted in a repeated recall for the same lesion and the confirmation of breast cancer. The average tumour size was 10.2 mm (range, 3−53 mm) at the first recall, compared to 17.3 mm (range, 4−88 mm) at the second recall (*P* < 0.001, Table 3). A majority of invasive cancers, namely 71.9% (64/89), was sized ≤10 mm at the first recall, compared to only 25% (22/89) of the cancers at the second recall (*P* < 0.001).

**Table 3:** Invasive cancers with a diagnostic delay following recall in cohort 2: tumour size measured at screening mammography.

	Measured at the screening mammogram with a false-negative assessment after first recall (*N* = 89)	Measured at a subsequent screening mammogram with breast cancer confirmation after second recall (*N* = 89)	*P* value
Mean tumour size, mm (range)	10.2 (3−53)	17.3 (4−88)	<0.001
Absolute tumour size, mm			
≤5 mm	14 (15.9)	1 (1.1)	<0.001
6−10 mm	49 (55.7)	21 (23.9)	
11−20 mm	20 (22.7)	42 (47.7)	
>20 mm	5 (5.7)	24 (27.3)	

### Variations among hospitals in delayed breast cancer confirmation after recall in cohort 2

The majority of the assessments after recall was performed in six regional hospitals located in our screening region. The proportion of women who experienced a more than 2-year diagnostic delay in their breast cancer confirmation varied from 0 to 4.8% among the hospitals (*P* = 0.027, Table 4). Hospitals II, III, IV and V are teaching hospitals that employ radiology residents, fellows and senior radiologists. The departments of radiology of hospitals I and VI are staffed by senior radiologists only.

**Table 4:** Delayed breast cancer confirmation after recall at screening mammography: variations among hospitals (diagnostic delay was defined as confirmation of breast cancer ≥ 24 months following initial recall).

Hospital	Delayed breast cancer confirmation, *N* (%)		Total, *N*
	Yes	No	
I	0 (0)	371 (100)	371
II	26 (2.8)	891 (97.2)	917
III	25 (3.0)	809 (97.0)	834
IV	29 (3.2)	878 (96.8)	907
V	12 (3.9)	294 (96.1)	306
VI	5 (4.8)	100 (95.2)	105
VII^a^	1 (1.4)	70 (98.6)	71
Total	98 (2.8)	3413 (97.2)	3511

^a^Remaining hospitals.

## Discussion

This study describes the frequency of a delay in breast cancer diagnosis after repeated recall at screening mammography, as well as the tumour characteristics of these cancers, the causes of diagnostic delay and the frequency of this delay among hospitals. Cohort 2 was characterised by a higher recall rate and cancer detection rate than cohort 1. Cohort 1 consisted of screen-film screens only, whereas the majority of women in cohort 2 was screened by digital mammography. In the Dutch screening programme, the transition from screen-film to digital screening significantly increased the recall rate and cancer detection rate, at the expense of a lower PPV of recall and biopsy.^17,18^ During two decades of screening mammography, we did not find a decrease in the proportion of recalled women who experienced a delay in breast cancer confirmation of at least 2 years. Most delays were caused by incorrect BI-RADS classifications at clinical assessment after recall. The tumour characteristics of breast cancers with a diagnostic delay were comparable to cancers without this delay, with the exception of the proportions of DCIS and tumour histology. The delayed confirmation of breast cancer significantly increased the mean tumour size and hospitals, where assessment of recalled lesions took place, showed significant variations in the proportion of women who experienced a diagnostic delay in the confirmation of their breast cancer.

We were surprised to find a comparable proportion in the two cohorts of women who faced a long delay in their breast cancer diagnosis, despite improvements in diagnostic imaging, biopsy procedures and patient management over the years. We previously found that most of the improvements in diagnostics and patient management after recall (e.g., breast MRI, SCNB, multidisciplinary meetings) were widely implemented after 2005 (cohort 2).^6^ We expected that the proportion of diagnostic delays among recalled women would be lower in the second decade because of these technical developments and continuously improving standards of care. Unfortunately, this was not the case. We did not use the transition from screen-film to digital screening mammography or the availability of two-view mammography at screening to define the cohorts as we feel that the reasons for delays in breast cancer confirmation after recall are primarily hospital related and not related to the screening procedure itself. Unfortunately, the limited number of delays did not allow us to perform a sophisticated time trend analysis using cohorts of, for example, 2 years each.

A few studies have previously reported on delayed breast cancer confirmation after recall at screening mammography, with 4.1−6.5% of women experiencing a diagnostic delay.^3,6,9^ These three studies, however, used different definitions for diagnostic delay. A majority of women had a diagnostic delay of 3−12 months, with only 4, 9 and 10 women respectively with a delay of at least 2 years. We found median delays of 26 and 25 months in the two study cohorts. Our lower proportion of 2.2−2.8% of diagnostic delays on average can be explained by the fact that we merely focused on delays of at least 2 years (until the next screening round) as these delays will probably have a more significant impact on survival and thus being more clinically relevant than shorter delays. Inclusion of shorter delay intervals would obviously increase and probably more than double our number of women with a delay in their breast cancer confirmation, but these delays were outside of the scope of the current study.

Most delays in cohort 2, which mainly comprised digital screening mammograms, were due to an incorrect interpretation of radiological findings after the first false-negative recall.

The exact impact of a delay in breast cancer confirmation on patient survival is a controversial topic, with studies yielding heterogeneous outcomes. Little is known on the natural history of breast cancer and researchers have raised the possibility of spontaneous breast cancer regression.^19,20^ This type of cancer may result in an infinite diagnostic delay as it may lead to recall and be overlooked, only to have vanished at a later screening and therefore go undetected. On the other hand, these cancers may be difficult to diagnose and may be the ones that are subject to delayed diagnosis. Over-diagnosis and subsequent over-treatment of cancers detected at screening mammography is another issue to be taken into account when dealing with diagnostic delays. Over-diagnosed breast cancers are cancers that would never have become clinical when not detected at screening and would never lead to death. While an infinite delay for these cancers is desirable, they may actually be difficult to diagnose and thus be included as cancers with a delay in diagnosis—and from a healthcare system view, we might well think that such delay should be avoided. Finally, we did not include interval cancers that were missed at the latest screen in this study as having a delayed diagnosis, since they do not participate in the next screening round. All in all, it is difficult to assess whether estimates from this study are likely to under- or over-estimate the actual occurrence of delay in diagnosis.^21–23^

Most studies focus on delayed diagnosis of symptomatic breast cancer and breast cancer in younger women, which differs from the asymptomatic breast cancers found at screening mammography in women aged over 50.^4,5,8,24^ We found a decrease in mean delay from 45 months to 33.8 months between the two cohorts and the majority of delays in cohort 2 were diagnosed at the next biennial screen, 2 years after the first, false-negative recall. However, it is likely that the delay of 33.8 months is somewhat flattered as cohort 2 had a shorter overall follow-up period.

The differences in tumour histology between cancers with or without a diagnostic delay in our study may have a multi-factorial explanation. Only a few diagnostic delay cases comprised DCIS. Improvement in biopsy techniques have made biopsy of calcifications faster, easier, and provides considerable amounts of tissue for pathological analysis.^25^ Moreover, the high inter-observer variation for the classification of microcalcifications at mammography may also encourage radiologists to rather biopsy than follow-up these lesions.^26,27^

We observed a shift in the distribution of mean tumour size among cancers with a long diagnostic delay. This phenomenon emphasises the need of a swift confirmation of breast cancer in order to prevent a further progression of the disease, as is stressed in other studies as well.^28–30^ Over the past two decades, screening mammography and clinical breast imaging have been subject to many changes, with the aim to improve the accuracy and efficiency of breast radiology. Despite the availability and proven benefit of state-of-the-art imaging, specialised outpatient clinics for breast care, specialised breast nurses and multidisciplinary team meetings and the continuous improvement of biopsy techniques, a delayed confirmation of breast cancer following recall at screening mammography continues to be a point of concern in the Netherlands and probably in other countries as well. An Italian biennial screen-film breast screening programme, conducted from 1992 to 2001, also reported a diagnostic delay in breast cancer diagnosis of more than 2 years in 2.1% of confirmed breast cancers, comparable to the 2.2% we observed in the era of screen-film screening.

Similar to screening mammography, screening programmes for other diseases have also shown benefit in terms of a reduction in cancer-related morbidity and mortality.^31–35^ In contrast to the screening mammography programme, the Dutch nationwide screening programmes for cervical cancer and large bowel cancer have embedded a quality assurance protocol with respect to structured assessment after recall. Quality assurance is partially based on accreditation, e.g., only accredited endoscopists are allowed to perform diagnostic colonoscopy after a positive screening result. Also, cervical smears are assessed in only a handful of accredited laboratories. Screening radiologists who are involved in the Dutch breast cancer screening programme have to obtain an accreditation by a national training programme prior to their employment as screening radiologists; they have to attend breast radiology courses at regular intervals and screening radiologists receive feedback on their performance continuously. These kinds of quality assurance measures are not mandatory at all for radiologists who perform the radiological assessment after recall. We found a worrisome variation in the proportions of delayed breast cancer cases among the hospitals handling the recalls in our screening region. Differences among hospitals may not be explained by the type of hospital (teaching versus non-teaching hospital) or case load, but probably by the experience of the clinical breast radiologists, as the lowest proportion of breast cancer delays was observed in a smaller non-teaching hospital. Moreover, there are similar diagnostic pathways due to national guidelines and no notable differences in diagnostic imaging capabilities. To our knowledge, no (recent) data are available on differences in diagnostic delay of breast cancer diagnosis in teaching versus non-teaching hospitals. Earlier studies on difference in breast cancer survival between teaching and non-teaching hospitals showed no statistically significant differences, although these results have to be interpreted with care as data on delayed diagnosis is not reported in these studies.^36–39^

To reduce the variations in diagnostic errors and delay an accreditation, similar to the one that is applied to screening radiologists, may be beneficial for radiology departments assessing recalled women and it could help to improve diagnostic accuracy, reduce diagnostic errors and prevent unnecessary delays. Perhaps, centralisation of breast care may also further improve the current standard of breast cancer diagnosis and treatment. Studies have shown that centralisation of complex and high-risk treatment in accredited hospitals may have a beneficial effect on the treatment outcome and survival.^40–43^ The use of breast assessment centres has also been studied and might be the first step towards centralisation of breast cancer care in the Netherlands.^24^ However, due to high patient volumes and concerns for the impact on demographics, centralisation of care in a limited number of hospitals in the whole country does not seem feasible. Nevertheless, cooperation between hospitals on a regional scale could prove beneficial, with implementation of adequate quality assurance, accreditation of radiologists handling referrals, and regular audits to continuously improve breast care in these hospitals.^24,44–46^

There are several strengths and limitations to this study. To our knowledge, this is the first study focusing on long delays in breast cancer confirmation after recall for a screen-detected mammographic abnormality. A large series of consecutive screening mammograms were included during two decades of screening mammography, with an almost complete follow-up of recalled women. On the other hand, certain outcome measures should be interpreted with care due to the relatively small sample size of patients with a delayed breast cancer diagnosis. Also, screening mammography programmes are constantly subject to changes and the design of the programmes and the assessment of recalled women differ between countries. These parameters influence diagnostic accuracy (both positively and negatively) and may limit extrapolation of our findings to other screening programmes.

We cannot rule out the rare occasion that a lesion with a delayed confirmation of malignancy was in fact precancerous rather than cancerous at the time of the initial assessment. However, the majority of delayed cases showed suspicious mammographic characteristics that should have been biopsied at initial workup. Moreover, in women with benign biopsy outcome at the initial workup, sampling errors will likely have caused the diagnostic delays as only benign pathological findings and no high-risk lesions, that are associated with an increased breast cancer risk in the future (e.g., flat epithelial atypia, atypical ductal hyperplasia or lobular carcinoma in situ), were reported at this workup.

In summary, we found that the proportion of women who experience a long delay in their breast cancer confirmation following recall at screening mammography did not decrease during 20 years of screening, despite improvements in diagnostic modalities, biopsy procedures and multidisciplinary approaches. These delays worsen the tumour size and may negatively influence prognosis of survival. Over-estimation of the negative impact on survival of a delayed breast cancer diagnosis may be due to over-diagnosed breast cancers and further study is needed on this subject. Further study is also necessary to determine whether accreditation of hospitals and/or centralisation of breast care may lower the number of diagnostic delays, especially in hospitals with larger proportions of women facing a diagnostic delay. We suggest that quality assurance not only covers the screening mammography programmes, but also the hospitals handling the workup of recalled women.

## Data Availability

Data are stored in a database maintained by one of the authors (L.E.M.D). The data are not publicly available. When data are needed, an official request can be made through the Department of Radiology of the Canisius Wilhelmina Hospital Nijmegen.
